# Supplementary material for: Decorin Secreted by Human Umbilical Cord Blood-Derived Mesenchymal Stem Cells Induces Macrophage Polarization via CD44 to Repair Hyperoxic Lung Injury
Source: Int J Mol Sci. 2019 Sep 27;20(19):4815. doi: 10.3390/ijms20194815 (PMC6801980; doi:10.3390/ijms20194815)
Supplement: Supplementary file 1 [file ijms-20-04815-s001.pdf]

Supplementary Materials:

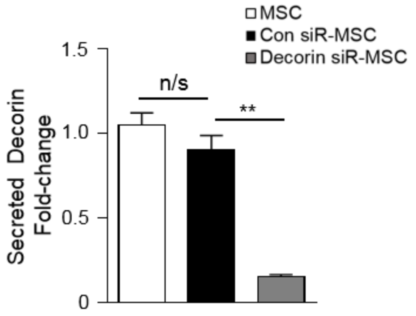

Figure S1. Silencing of decorin expression in MSCs. MSCs were transfected with 100 nM of scramble siRNA (Con siR-MSC) or decorin siRNA (Decorin siR-MSC) for 24 hours. MSCs transfected of decorin siRNA secreted the suppressed decorin secretion. Supernatants were analyzed for decorin secretion of MSCs by ELISA. Expression levels were normalized to MSC alone, which was defined as 1. The data are presented as mean ± SD, n=3 per group. **p < .01, * p < .05, n/s; not significant.

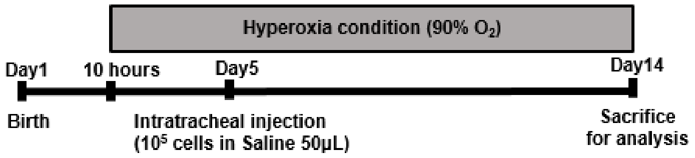

Figure S2. Scheme for a hyperoxic lung injury rat model. Rat pups were raised on hyperoxia condition for 14 days after birth. Intratracheal injection of 1 x 10^5 MSCs have been taken on day5. Damaged lung tissues and BALF were isolated and analyzed at P14.

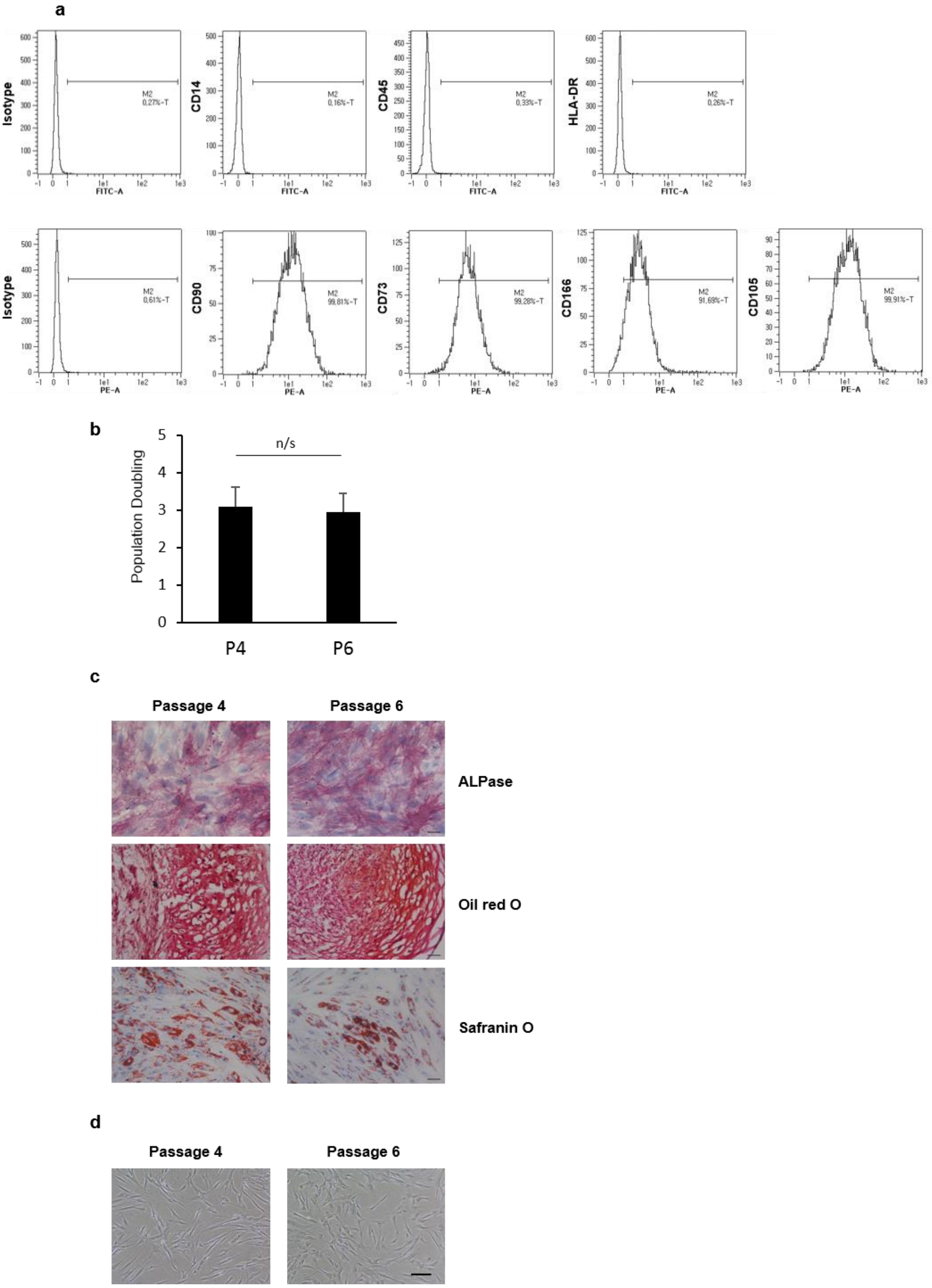

Figure S3. Characteristics of MSCs. MSCs were isolated from donors. (**a**) MSCs were positive to CD90, CD73, CD166 and CD105 (≥90 %). CD14, CD45, and HLA-DR MSC markers were negative (≤1 %). (**b**) Cell growth rate for passage 4 and 6 of MSCs was determined by population doubling. (**c**) The differentiation capacity is determined by osteogenic (ALPase staining), chondrogenic (Safranin O staining), and adipogenic (Oil red O) differentiation. Scale bar = 100 μm. (**d**) Cellular senescence of MSCs in passage 4 and passage 6 was determined by senescence-associated beta-galactosidage (SA-β-gal). The data are presented as mean ± SD, n=3 per group. **p < .01, * p < .05, n/s; not significant.

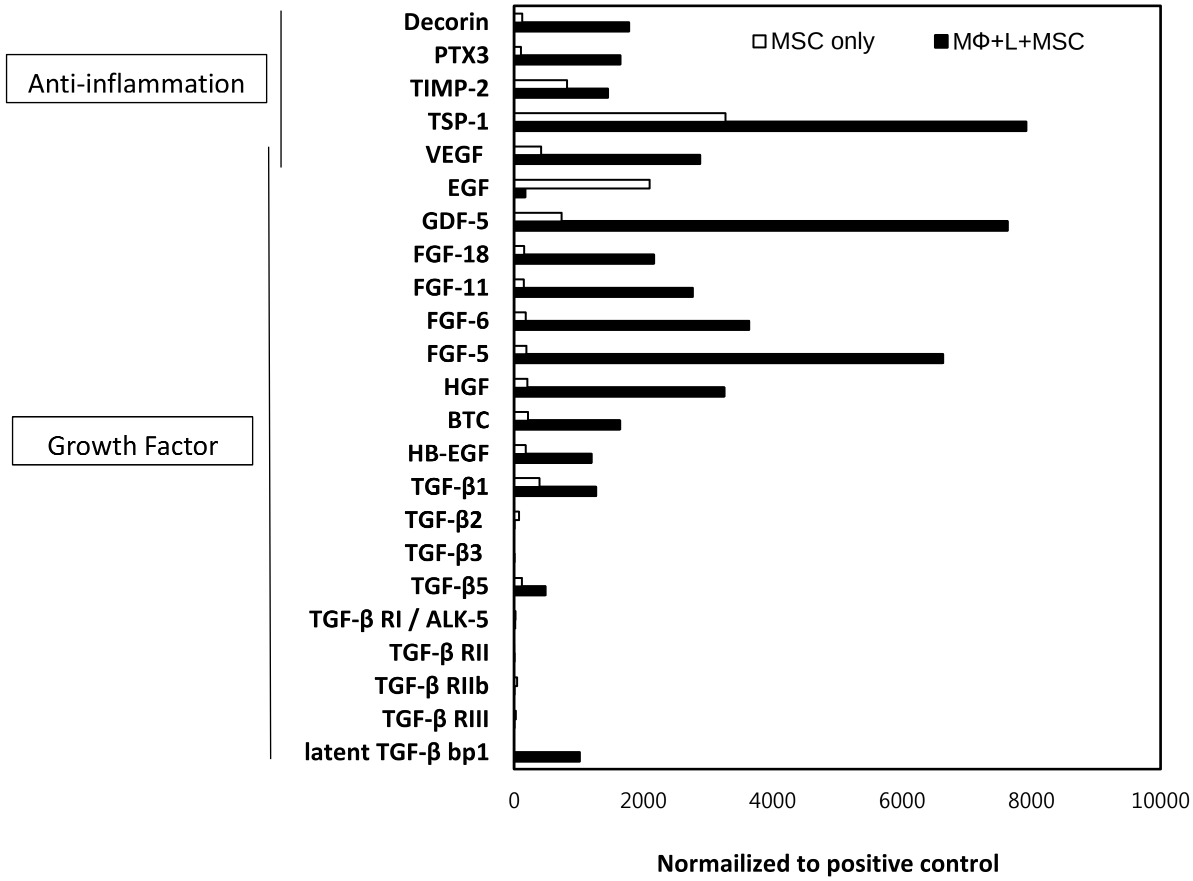

Figure S4. Secretome analysis of MSCs under inflammation conditions. Biotin label-based antibody array was performed using the supernatants collected from MSC only and MSC with LPS-stimulated macrophages. The quantification of optical density was analyzed and normalized to the positive control.

Supplemental method:

Biotin Label-based Human Antibody Array

The proteins secreted from MSCs only and MSCs with LPS-stimulated macrophages were detected by the Human Antibody Array L series (RayBiotech, Peachtree Corners, GA, USA). The supernatants from each sample was applied to a glass chip containing 507 antibodies followed by manufacturer's instructions. Fluorescence detection were performed using a GenePix 4000B scanner (Molecular Devices, Sunnyvale, CA). The images were analyzed by E-biogen (Seoul, Korea) using GenePix Pro 6.0 software (Axon Instruments). The quantified values were normalized to the positive control.
